# Supplementary material for: ProLonged Liposomal Delivery of TLR7/8 Agonist for Enhanced Cancer Vaccine
Source: Vaccines (Basel). 2023 Sep 19;11(9):1503. doi: 10.3390/vaccines11091503 (PMC10538091; doi:10.3390/vaccines11091503)
Supplement: Supplementary file 1 [file vaccines-11-01503-s001.zip › vaccines-2554315-supplementary.pdf]

vaccines

Supplementary Materials

ProLonged Liposomal Delivery of TLR7/8 Agonist for Enhanced Cancer Vaccine

Sehui Kim [1,2,†], Yeji Park [1,2,†], Jeonghun Kim [1], Kyungmin Choi [1], Sohyun Kim [1], Taegyun Kang [1], Inho Lee [1] Yong Taik Lim [2], Soong Ho Um [1,3] and Chul Kim [1,*]

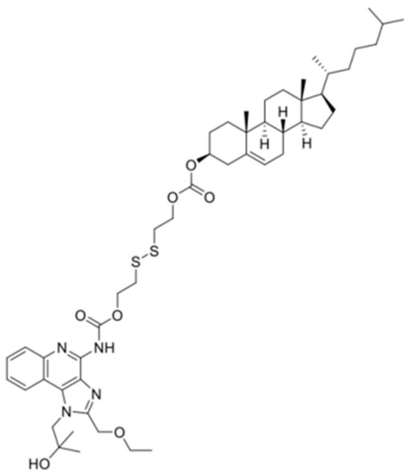

Supplementary Figure S1. Structure drawing of ProLNG-S. Active site of resiquimod (R848) is sealed by cholesterol, it gives ease of formulation and enhance safety of materials. The fabrication method of ProLNG-S was explained in previous article [1].

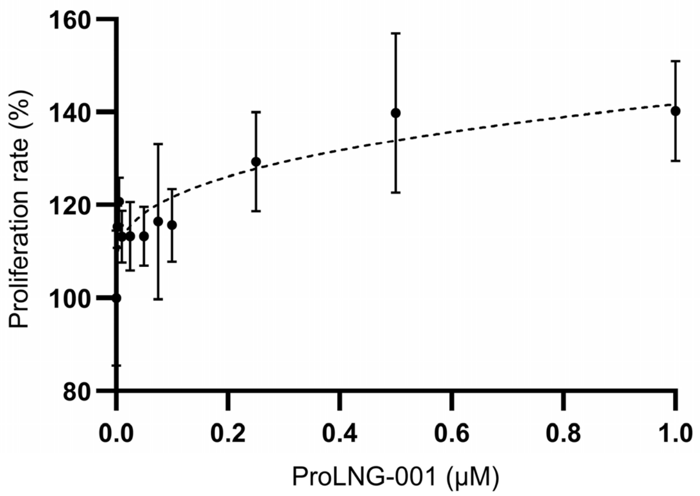

Supplementary Figure S2. *In vitro* BMDCs proliferation assay. Cell proliferation of BMDCs with indicated concentration of ProLNG-001 was measured by an MTS assay after 24 hours.

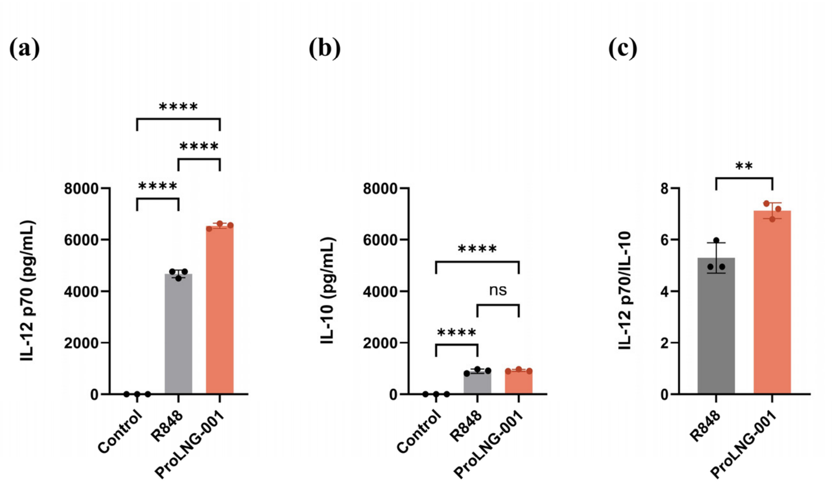

Supplementary Figure S3. *In vitro* cytokine production in BMDCs. R848 0.5 μg or ProLNG-001 with the equivalent amount of R848 were treated to BMDCs for 24 hours. The supernatant was collected and (a) IL-12 (p70), and (b) IL-10 production was measured by ELISA. (c) The ratio of IL-12 (p70) to IL-10 (n=3).

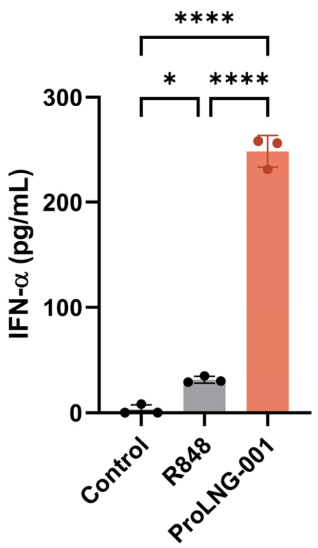

Supplementary Figure S4. *In vitro* IFN-α production in mouse pDCs. R848 1 μg or ProLNG-001 with the equivalent amount of R848 were treated to pDCs and incubated for 24 hours. The supernatant was collected and IFN-α production was measured by ELISA (n=3).

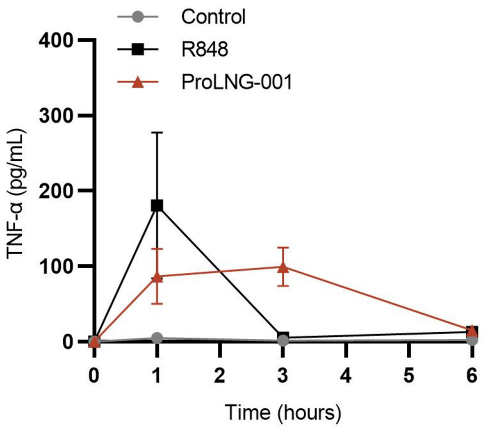

Supplementary Figure S5. TNF-*α* level by time in mouse plasma. C57BL/6 mice were subcutaneously injected with PBS, resiquimod (R848) 25 µg, ProLNG-001 140 µg. At 0, 1, 3, and 6 hours after injection, mice plasma was collected. ELISA was performed to confirm the TNF-*α* concentration in the plasma.

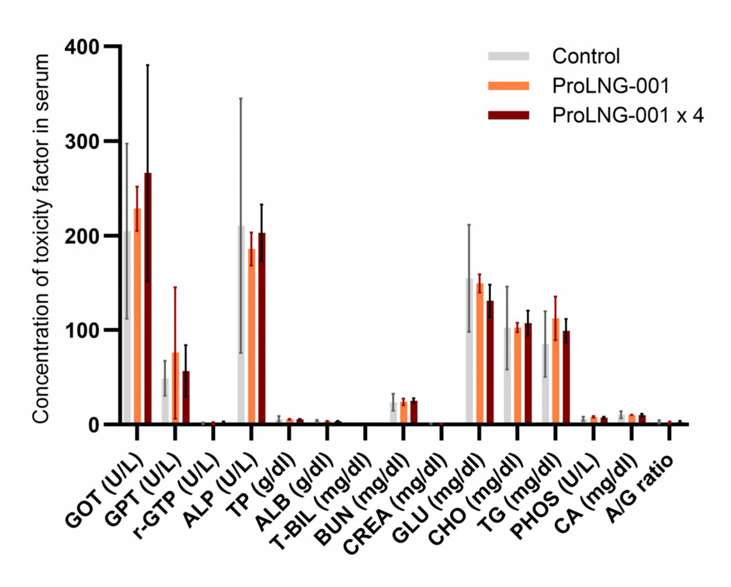

Supplementary Figure S6. Repeated toxicity test in mouse. C57BL/6 mice were subcutaneously injected with PBS, ProLNG-001 140 μg, ProLNG-001 560 μg (n=5/group). Each group injected the sample 4 times at a week interval and collected plasma two weeks after the last injection. Factors related to liver and kidney toxicity were identified through blood biochemical tests.

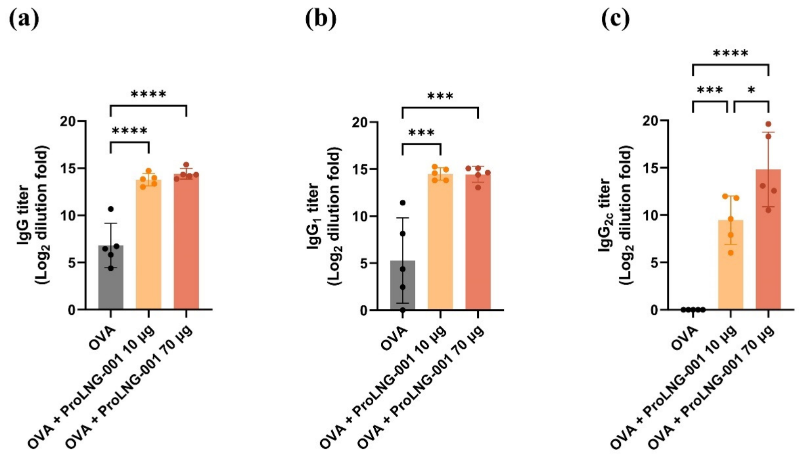

Supplementary Figure S7. IgG production in C57BL/6 immunized by OVA+ProLNG-001. C57BL/6 were vaccinated with OVA 10 μg, OVA 10 μg + ProLNG-001 10 μg, and OVA 10 μg + ProLNG-001 70 μg via two subcutaneous injections at 2-week intervals on the upper right back. Mice plasma was collected two weeks after the last vaccination, and plasma was serially diluted for measuring IgG titer. (a)Total IgG, (b)IgG1, and (c)IgG2c against OVA protein were profiled (n=5).

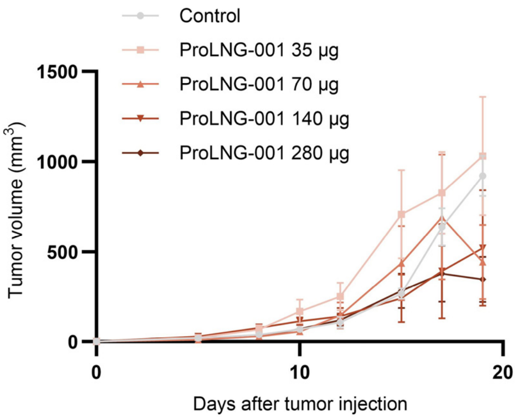

Supplementary Figure S8. Anti-tumor effects depending on the various doses of ProLNG-001 in the B16-OVA tumor-bearing mouse model. C57BL/6 mice were subcutaneously injected with 3 × 10^5 B16-OVA cells on the upper right back. On days 3 and 10 after tumor inoculation, subcutaneous injections of 35 μg, 70 μg, 140 μg, and 280 μg of ProLNG-001 were administered. Tumor growth was measured at least every two or three days. Tumor growth curves are shown (n = 3/group).

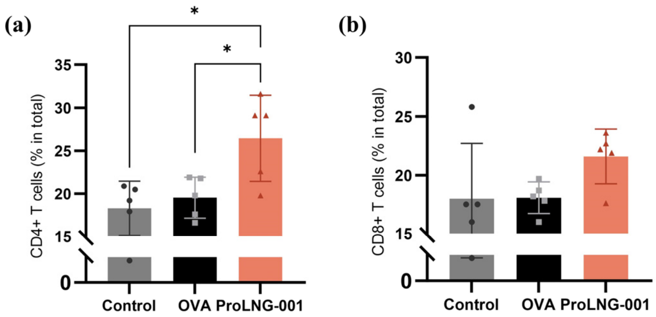

Supplementary Figure S9. Increasement of CD4+ T cells and CD8+ T cells in mouse spleen. CD4+, CD8+ T cells were analyzed by flow cytometry in ProLNG-001/OVA immunized mouse model. C57BL/6 mice were injected two times with a week interval with PBS (control), OVA protein (10 μg) and ProLNG-001 (140 μg) + OVA (10 μg).

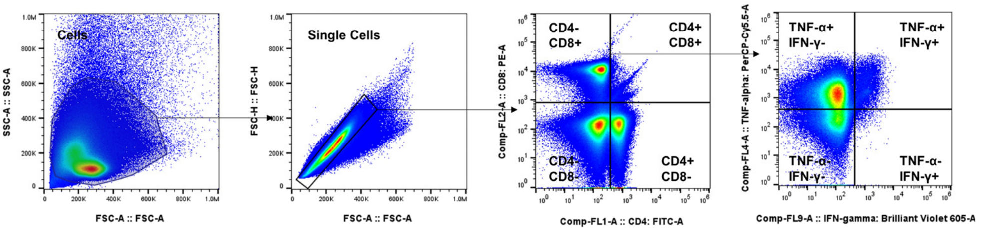

Supplementary Figure S10. Representative flow cytometer gating strategy of cytokine secreting T cells in the spleen. This experiment was performed in **Figure 5**, and specific cells were isolated using CD4, CD8, TNF-α, and IFN-γ markers.

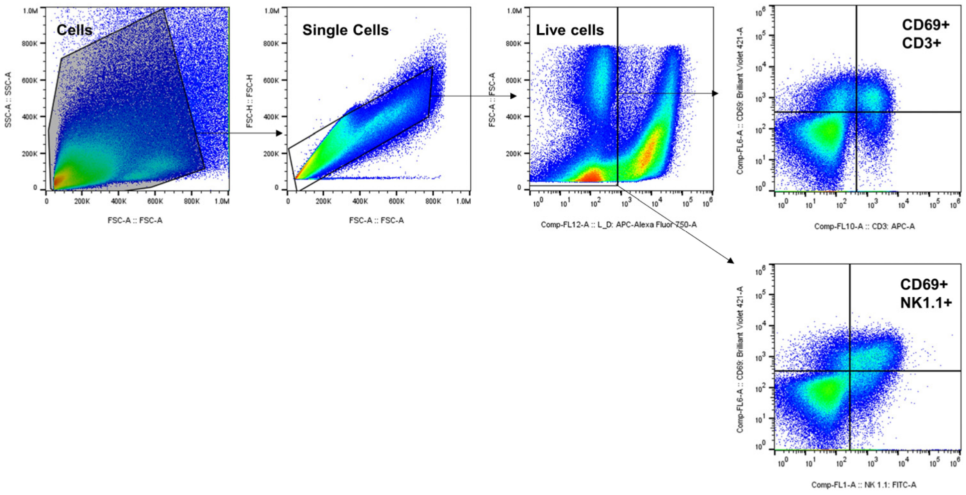

Supplementary Figure S11. Representative flow cytometer gating strategy of cytokine secreting T cells in the tumor. This experiment was performed in **Figure 5**, and specific cells were isolated using CD3, NK1.1, and CD69 markers.

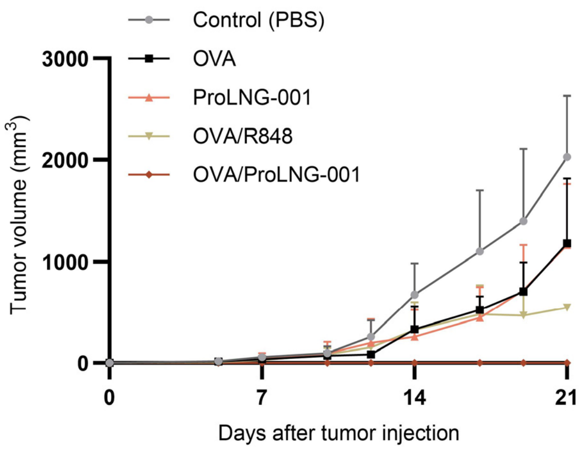

Supplementary Figure S12. Growth of B16-OVA melanoma tumors of immunized C57BL/6 mice in the prophylactic model. C57BL/6 mice were injected two times by 3 weeks interval with PBS (control), OVA protein (10 µg), ProLNG-001 (70 µg), OVA (10 µg) + R848 (25 µg), and OVA (10 µg) + ProLNG-001 (70 µg). A week after the last immunization, 3×10^5 B16-OVA cells were inoculated on the upper right back, and tumor growth was measured at least every two or three days (n = 3/group).

References

1. Jin, S.M.; Yoo, Y.J.; Shin, H.S.; Kim, S.; Lee, S.N.; Lee, C.H.; Kim, H.; Kim, J.-E.; Bae, Y.-S.; Hong, J.; et al. A Nanoadjuvant That Dynamically Coordinates Innate Immune Stimuli Activation Enhances Cancer Immunotherapy and Reduces Immune Cell Exhaustion. *Nat. Nanotechnol* **2023**, *18*, 390–402, doi:10.1038/s41565-022-01296-w.
